# An occupational health intervention programme for workers at high risk for sickness absence. Cost effectiveness analysis based on a randomised controlled trial

**DOI:** 10.1136/oem.2007.033167

**Published:** 2007-10-12

**Authors:** S Taimela, S Justén, P Aronen, H Sintonen, E Läärä, A Malmivaara, J Tiekso, T Aro

**Affiliations:** 1 Evalua International, Vantaa, Finland; 2 University of Helsinki, Department of Public Health, and FinOHTA, Helsinki, Finland; 3 University of Oulu, Department of Mathematical Sciences, Oulu, Finland; 4 Finnish Office for Health Technology Assessment, FinOHTA/Stakes, Helsinki, Finland; 5 Mutual Pension Insurance Company Ilmarinen, Helsinki, Finland

## Abstract

**Objectives:**

To determine whether, from a healthcare perspective, a specific occupational health intervention is cost effective in reducing sickness absence when compared with usual care in occupational health in workers with high risk of sickness absence.

**Methods:**

Economic evaluation alongside a randomised controlled trial. 418 workers with high risk of sickness absence from one corporation were randomised to intervention (n = 209) or to usual care (n = 209). The subjects in the intervention group were invited to occupational health service for a consultation. The intervention included, if appropriate, a referral to specialist treatment. Register data of sickness absence were available for 384 subjects and questionnaire data on healthcare costs from 272 subjects. Missing direct total cost data were imputed using a two-part regression model. Primary outcome measures were sickness absence days and direct healthcare costs up to 12 months after randomisation. Cost effectiveness (CE) was expressed as an incremental CE ratio, CE plane and CE acceptability curve with both available direct total cost data and missing total cost data imputed.

**Results:**

After one year, the mean of sickness absence was 30 days in the usual care group (n = 192) and 11 days less (95% CI 1 to 20 days) in the intervention group (n = 192). Among the employees with available cost data, the mean days of sickness absence were 22 and 24, and the mean total cost €974 and €1049 in the intervention group (n = 134) and in the usual care group (n = 138), respectively. The intervention turned out to be dominant—both cost saving and more effective than usual occupational health care. The saving was €43 per sickness absence day avoided with available direct total cost data, and €17 with missing total cost data imputed.

**Conclusions:**

One year follow-up data show that occupational health intervention for workers with high risk of sickness absence is a cost effective use of healthcare resources.

Sickness absence, defined as non-attendance by an employee at work due to a health complaint, places a major economic burden on employers, the healthcare system and society as a whole. The optimal occupational health intervention strategy for employees with high risk of sickness absence remains uncertain. Evidence from a few randomised trials^1^ ^2^ suggests that specific intervention programmes targeted at selected individuals at high risk of sickness absence may provide benefits. It is not, however, clear whether occupational health intervention in conjunction with identification of high-risk employees can generate similar benefits for employers and employees and also be a cost effective use of healthcare resources.

A Finnish trial^3^ used a health survey in order to identify subjects with a high risk of sickness absence. Subsequently, employees in this subgroup were randomised into additional occupational health intervention or to usual care at occupational health. The results showed a clear difference in favour of the intervention arm in the days of sickness absence, the primary outcome measure: on average 11 days for the one-year follow-up period. The difference in sickness absence was of such magnitude that is likely to be economically advantageous, but the cost consequences need to be considered in order to evaluate the cost effectiveness of the targeted occupational health intervention in comparison with usual care in the high-risk group. We report such an economic evaluation conducted prospectively alongside the Finnish occupational health intervention trial.

## METHODS

Full details of the randomised controlled trial have been published previously.^3^ In brief, the trial was conducted to compare the differences between the occupational health intervention programme and usual care for employees at high risk of sickness absence at 12 months. The subjects came from one corporation in Finland. 49% of them were employed in the construction industry (civil engineering, building contracting, technical building services and building materials industry). The remaining 51% were employed in the repair, service and maintenance of buildings, industrial installations, or communications networks.

At the beginning of the study the employees (n = 1341; 88% males; 62% blue-collar) were divided into three study groups “low risk” (n = 386), “intermediate risk” (n = 537) and “high risk” (n = 418) of sickness absence on the basis of a self-administrated questionnaire with a priori defined interpretation cut-off limits. Subjects who reported problems with future working ability, pain, impairment due to musculoskeletal problems, insomnia or insufficient sleep, frequent stress or fatigue, or had a high depression score, were classified into the high risk group. Of the employees who met the trial eligibility criteria, 209 were randomised to the occupational health intervention and 209 to the control group receiving usual care at occupational health. The interventions were provided between October 2004 and September 2005. At baseline and 12 months, employees completed the questionnaire, which was used to estimate self-rated health problems and working ability.^4^ At 12 months they also completed a questionnaire concerning healthcare resource use during the past 12 months.

### Intervention

The employees in the targeted occupational health intervention group attended the occupational health programme operated by their own occupational nurses and physicians. They received personal feedback of their survey results and an invitation to a consultation at their local occupational health service (OHS). The main purpose of the consultation was the construction of an action plan, and if appropriate, referral to a further consultation by a specialist or psychologist. The visits had a predefined content including procedures on how to further diagnose diseases and rules for further actions according to the process description. The occupational nurse compiled a personal file for each employee in the intervention group, which included information about the treatments and health advice received at the OHS, the referrals to further evaluation or interventions, the considerations of OHS professionals that no further actions were needed, and the refusals of some employees to take further action. 142 (68%) subjects participated in the OHS intervention. Forty eight occupational health centres were involved in the intervention programme.

The employees in the control group could consult their occupational nurse or physician on request, but they did not get feedback of their health survey results and were not invited for a consultation.

### Outcome measures

Effectiveness was measured primarily by the difference in sickness absence days between the two groups at follow-up. Employee-specific sickness absence data, without medical diagnosis, were obtained from the employer’s records, covering the period from 1 October 2004 to 30 September 2005.

In a secondary analysis effectiveness was measured by difference in self-rated health outcomes at follow-up in order to analyse how the differences in sickness absence between the treatment arms were related to perceived health and working ability. At 12 months follow-up, employees were asked in a postal survey about sleep disturbances, work-related stress and fatigue, depression, pain, disability due to musculoskeletal problems, their conception of future working ability, and changes in their working ability during the past year.

### Resource use

In the postal survey at 12 months the employees were also requested to complete a questionnaire concerning healthcare resource use during the past 12 months. Table 1 provides an overview of the resource use, unit costs,^5^ ^6^ and mean costs by items of resource use, and the mean direct total costs (direct healthcare costs and travelling costs, which can be regarded as an item of direct non-healthcare costs) based on the subjects, from whom the resource use data were available. To avoid double counting, productivity costs were not included, as they arise from sickness absence days, which was the primary outcome of the study. All costs are expressed in euros at the 2004 price level.

**Table 1:** The items of resource use, their unit costs, amount of resource use, mean costs (standard deviations) and associated total costs based on the subjects for whom the resource use data were available

Variable	Description	Unit cost (€)	Total usage (number of visits)		Mean (SD) costs (€)	
			Intervention (n = 134)	Control (n = 138)	Intervention (n = 134)	Control (n = 138)
UH_days	Number of in-patient days at university hospital	636.1	29	24	138 (695)	111 (420)
OHC_doc	Doctor visits in occupational health care (OHC)	41.2	419	495	129 (128)	148 (156)
UH_OC	Visits at outpatient clinic in a university hospital	202.8	69	47	104 (185)	69 (126)
CH_days	Number of in-patient days at central hospital	451.6	26	35	88 (438)	115 (578)
CH_OC	Visits at outpatient clinic in a central hospital	183.2	49	30	67 (363)	40 (220)
Oth_phys	Physiotherapist visits outside OHC	30.0	272	236	61 (75)	51 (92)
OHC_nur	Nurse visits in OHC	22.7	296	235	50 (93)	39 (60)
OHC_phys	Physiotherapist visits in OHC	49.7	134	123	50 (194)	44 (121)
Rehab_days	Number of in-patient days at rehabilitation	112.7	55	69	46 (274)	56 (323)
RH_OC	Visits at outpatient clinic in a regional hospital	179.8	29	53	39 (214)	69 (145)
PH_days	Number of in-patient days at private hospital	510.2	9	1	34 (355)	4 (43)
PH_GP	General practitioner visits in public healthcare	65.6	67	76	33 (80)	36 (66)
Oth_Cons	Visits at other private consultant	79.6	44	53	26 (64)	31 (101)
Orthop	Visits at private orthopaedic consultant	121.0	26	32	23 (82)	28 (117)
OHC_other	Visits at other healthcare professionals in OHC	33.9	60	46	15 (70)	11 (44)
RH_days	Number of in-patient days at regional hospital	400.2	5	24	15 (91)	70 (407)
PH_nur	Nurse visits in public healthcare	30.5	36	46	8 (25)	10 (55)
Priv_GP	General practitioner visits in private healthcare	57.9	18	36	8 (40)	15 (51)
CHCW_days	Number of in-patient days at community health centre	148.0	6	0	7 (77)	0 (0)
MHC	Visits at mental health clinic	108.1	8	10	6 (47)	8 (28)
Oth_OC	Visits at other hospital outpatient clinics	191.2	4	3	6 (211)	4 (212)
Tel_adv	Telephone health advice	17.4	38	83	5 (20)	10 (26)
UH_n	Number of visits at university hospital	30.9	19	20	4 (20)	4 (17)
CH_n	Number of visits at central hospital	30.9	18	17	4 (22)	4 (14)
Psych	Visits at private psychiatrist or psychologist	88.1	6	75	4 (39)	48 (453)
RH_n	Number of visits at regional hospital	30.9	9	34	2 (14)	8 (46)
Rehab_n	Number of visits at in-house rehabilitation centre	13.2	9	15	1 (4)	1 (12)
OthH_n	Number of visits at other hospital	6.0	7	2	0 (3)	0 (1)
PH_n	Number of visits at private hospital	13.2	1	3	0 (1)	0 (2)
OthH_days	Number of in-patient days at other hospitals	510.2	0	4	0 (0)	15 (137)
CHCW_n	Number of visits at community health centre ward	6.0	0	1	0 (0)	0 (1)
	Mean direct total cost				974	1049
	Total costs				130469	144741

The source for the unit costs was Hujanen^5^ for all other items except Kansaneläkelaitos^6^ for “Rehab_days”. The unit costs are expressed in euros at the 2004 price level.

### Statistical methods

Analyses were performed according to the intention-to-treat principle. Analysis of variance and covariance (ANCOVA) was used in the comparison of the mean costs and sickness absence between the groups. Despite the heavily skewed distributions of these variables, in large samples conventional estimation methods based on linear regression models are expected to provide reasonably valid results.^7^

Missing total direct cost data were imputed using a two-part regression model with employee characteristics at baseline and sickness absence days during the follow-up as explanatory variables. Employee characteristics used as covariates were: age, gender, body mass index, working status (white- or blue-collar), number of health problems (comorbidity), alcohol consumption, physical impairment at work, pain score, insufficient sleep, and daytime sleepiness. We explored five techniques for imputation:^8^^–^10 ordinary least squares (OLS), lognormal, gamma and median regression, and multiple imputation (MICE package). The first four were applied alone and in combination with logistic regression (logit) when a two-part method was used. A priori, a two-part approach was expected to be most appropriate due to the fact that typically different processes determine the probability of incurring any cost (seeking care) and the amounts of costs eventually incurred. The validity of imputations was explored by dividing the observations with full data into two groups and using one of them to produce the prognoses and another for validation. The predicted accuracy was assessed by using root mean square error, bias, mean squared prediction error and mean absolute prediction error. We chose the estimates based on logit with OLS, because they performed best in the accuracy tests (data not shown) and were conservative in comparison to those produced by other methods. Thus, in the first part of the imputation model, logistic regression was used to predict whether the employee had any costs or not. In the second part of the model, OLS regression was used to predict the direct total cost for those who had incurred any cost. Then these two predictions were multiplied to impute a cost estimate for those with missing cost data. The results are reported both with available total cost data and missing total costs imputed.^10^

To assess uncertainty, one-way (cost variables plus or minus 50% of base value) and probabilistic (bootstrapping with 10 000 replicates) sensitivity analyses were carried out. The latter were performed for both observed and imputed total cost data. Results are given as a tornado diagram, mean incremental costs and effects with their 95% confidence intervals, incremental cost effectiveness ratio (ICER), cost effectiveness plane, and cost effectiveness acceptability curve (CEAC).^10^

## RESULTS

Register data of sickness absence after 12 months of follow-up were available for 192 (92%) subjects in the intervention group, and 192 (92%) subjects in the control group: the employment of 17 subjects had terminated during the follow-up in both groups. Questionnaire data on healthcare costs and self-rated health problems were available from 134 subjects (64%) in the intervention group and 138 (67%) subjects in the control group. Table 2 shows the mean sickness absence days and the mean (SD) total direct costs in the groups, subdivided by whether the resource use data were available or missing.

**Table 2:** The mean number of sickness absence days (register data) at baseline and at follow-up as well as the mean and standard deviation (SD) for direct total costs

Availability of resource use data	Study group	Group size	Sick leave days per group						Direct total cost			
			Baseline			Follow-up						
			% zero	Mean	SD	% zero	Mean	SD	% zero	Mean	SD	
Data available	Control	138	36	16.9	37	25	23.7	45	4	1048.9	1368	
	Intervention	134	29	18.2	34	32	22.0	50	4	973.6	1628	
Data missing	Control	54	31	17.4	29	19	45.8	67	4	1261.3	1183	*
	Intervention	58	21	15.4	29	29	13.1	25	3	813.0	746	*
All	Control	192	34	17.1	35	23	29.9	53	4	1108.6	1319	†
	Intervention	192	27	17.4	32	31	19.3	44	4	925.1	1420	†

*Direct cost data are based on missing data imputation.

†Direct cost data are partly based on missing data imputation.

The results are presented by randomised groups among those subjects for whom the resource use questionnaire data were available, resource use data were missing, and for all subjects.

### Healthcare use

Table 1 shows the use of healthcare resources by the groups. The mean direct total cost using the available cost data was €974 (range €0–€11 501; quartiles: €184, €399, €939) and €1049 (range €0–€6908; quartiles: €165, €507, €1340) in the intervention (n = 134) and control (n = 138) group, respectively. With the missing direct total costs imputed the mean was €925 (range €0–€11,501; quartiles: €213, €525, €955) and €1109 (range €0–€6908; quartiles: €250, €625, €1382) in the intervention (n = 192) and control (n = 192) group, respectively.

### Cost effectiveness analyses

The intervention turned out to be dominant—that is, both cost saving and more effective than usual occupational health care. The saving was €43 and €17 per sickness absence day avoided by using the available direct cost data and data with missing total costs imputed, respectively.

**Figure 1:**
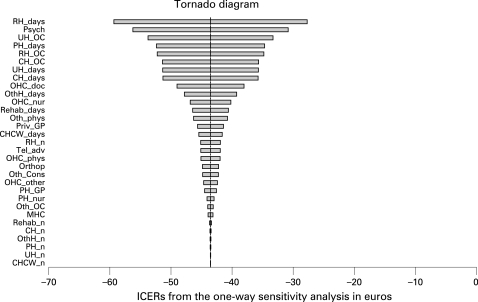
Tornado diagram showing the influence of changing values of any variable (SD 50%) on incremental cost effectiveness ratio (ICER) when other variables remain in their base values. In the graph variables are ranked on the basis of their influence (the most influential variable is on the top). The meanings of the abbreviations are shown in the table 1.

**Figure 2:**
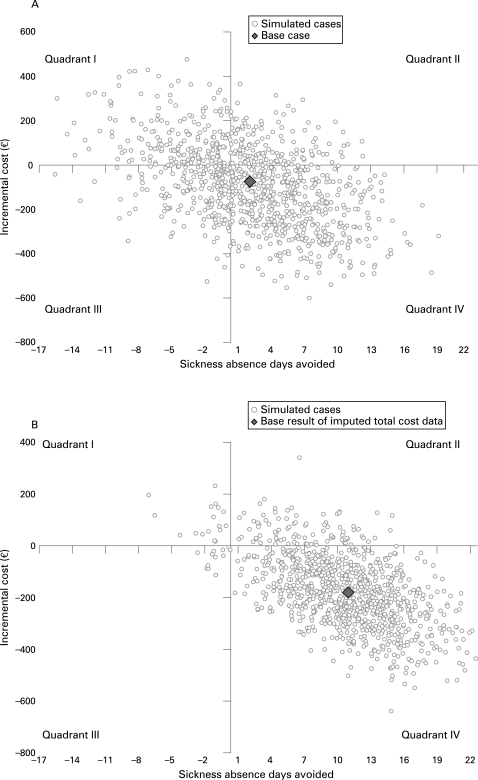
Cost effectiveness planes. Base case results indicated by a diamond. (A) With cost data based on the survey responses. In 49.9% of simulated cases intervention was both cost saving and more effective (Quadrant IV), in 17.7% cost saving and less effective (Quadrant III), in 12.5% more costly and more effective (Quadrant II), and in 19.9% of cases more costly and less effective (Quadrant I). (B) With missing cost data imputed. In 89.5% of simulated cases intervention was both cost saving and more effective (Quadrant IV), in 0.9% cost saving and less effective (Quadrant III), in 8.7% more costly and more effective (Quadrant II), and in 0.9% of cases more costly and less effective (Quadrant I).

**Figure 3:**
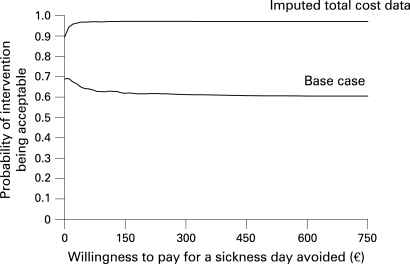
Cost effectiveness acceptability curves with total cost data based on questionnaire responses (base case analysis), and total cost data with imputed missing values (imputed total cost data).

With available total cost data from the 272 subjects (and sickness absence data from the same subjects) one-way sensitivity analyses revealed that the result was not sensitive to any cost variables: the incremental cost-effectiveness ratio varied from −€59 to −€28 per sickness absence day avoided—that is, in all cases the intervention was dominant (fig 1). The probabilistic sensitivity analysis showed that the mean incremental cost was −€80 (95% CI −429 to +290) and the mean incremental effect 1.8 days (95% CI −9.7 to +12.4) of avoided work absence. The cost effectiveness plane suggested that for about half of the simulated cases the intervention was cost saving and more effective (fig 2A). At any level of societal willingness to pay for a sickness absence day avoided the probability of the intervention being acceptable is around 60–70% (fig 3).

With available total cost data from the 272 subjects and missing total cost data imputed for 112 subjects (and sickness absence register data from all the 384 subjects) the probabilistic sensitivity analysis resulted in a mean incremental cost of −€180 (95% CI −452 to +98) and a mean incremental effect of 10.5 days (95% CI 0.6 to +20.4) of avoided work absence. According to the cost effectiveness plane in over 90% of the simulated cases the intervention was cost saving and more effective (fig 2B). If the societal willingness to pay for a sickness absence day avoided was, for example, €60 then the probability that the intervention is acceptable would be 97% (fig 3).

### Health outcomes

Table 3 shows the self-rated health outcomes by the groups. No difference was found in self-rated working ability, or perceived change in working ability between the groups. Neither was any difference found in the prevalence of distinct health problems (depression, fatigue, stress, pain, physical impairment, insomnia) between the groups. However, depression, fatigue and stress tended to be more prevalent in the control group than in the intervention group at 12-month follow-up. If the effectiveness of intervention is measured in terms of health outcomes, the intervention seems to be weakly dominant—that is, it produces the same effectiveness at a lower cost.

**Table 3:** Changes in working ability and the presence of health problems in the treatment arms at the end of the follow-up

	Control (n = 138)	Intervention (n = 134)	95% CI of the difference
Change in working ability (%)			
Much better	1	4	
Slightly better	13	11	
No change	47	49	
Slightly worse	31	31	
Much worse	8	6	
Presence of health problems (%)			
Depression	14	8	−3 to 14
Fatigue	8	4	−2 to 11
Stress	7	2	−1 to 11
Pain	19	20	−11 to 9
Physical impairment	58	51	−5 to 19
Insomnia	11	11	−8 to 8
Working ability	49	52	−15 to 9
Depression OR fatigue OR stress	19	11	−1 to 17

## DISCUSSION

### Main findings

The intervention, a personal feedback of the health survey results and an invitation to a consultation at the local OHS, was cost-saving and more effective in reducing sickness absence than usual occupational health care (dominant in terms of health economics^10^). The cost-effectiveness was not sensitive to any cost variables and the result did not change whether we used available cost data or data where missing total costs were imputed. The health survey outcome measures showed that the differences between the two groups were minimal concerning the subjects’ self-rated health problems. In other words, the intervention produced the same (self-rated) health outcomes at a lower cost than usual care.

### Strengths and weaknesses of the study

Our primary outcome was based on register data on sickness absence, which has several advantages: good coverage, accuracy and consistency.^11^ We were able to collect sickness absence data for all employees, whose employment still continued at the 12-month follow-up time point (n = 192 in both groups). The resource use data were collected by a questionnaire in a postal survey, and we received 134 responses in the intervention group and 138 responses in the usual care group. The overall response rate of about 70% in a postal survey is reasonably high. Comparison of the number of sickness absence days between the intervention and usual care (control) groups among employees with complete data and among employees with available cost data indicated however that the total cost data of employees were not missing completely at random (table 2). It appeared that the non-respondents in the usual care (control) group had significantly more sickness absence than the respondents, while such difference between the respondents and non-respondents in the intervention group was not found. This difference may be a side-effect of the intervention: participation in the intervention may have reduced the usual non-responding tendency among the most high-risk subjects. Therefore imputation of missing total cost data was deemed necessary to produce less biased cost-effectiveness results. A two-part approach to imputation was chosen due to the fact that typically different processes determine the probability of seeking care and the amounts of costs eventually incurred. Several regression techniques were explored in part two of the two-part approach. We chose the results based on OLS regression, because it performed best in the accuracy tests and produced conservative estimates in comparison with other methods. Therefore the imputed total costs are more likely underestimates rather than overestimates of the real total costs.

One-way sensitivity analyses revealed that the result was not sensitive to any cost variables. The cost effectiveness acceptability curves indicate that when the uncertainty both on the cost and effectiveness side is accounted for in the probabilistic sensitivity analysis, the intervention is acceptable in about 70% of cases at all levels of willingness to pay for a sickness absence day avoided in the base case, in which only data from the subjects for whom the direct total cost were available were used. With missing total costs imputed the acceptability rises to well over 90% at all levels of willingness to pay. As the number of sickness days was observed to increase from the baseline less in the intervention group than in the control group, it is probable that the use of health services and thus costs would also increase less, making the cost difference between the groups larger. Therefore, the almost 70% acceptability in the base case probably represents a “worst case scenario” and is already an indication of the superiority of the intervention. Using the whole set of the register data on sickness absences, and imputing the missing total cost data, probably leads to the least biased estimate of the acceptability (around 90%), even though imputation does bring some uncertainty around this estimate.

The health outcomes were based on available data, as imputation was not feasible. Therefore these results should be interpreted with caution as the non-responding to the postal survey was not completely at random. Missing data may have biased the health-related results against the intervention group.

### Some differences compared with previous studies

Systematic reviews of (randomised) trials on occupational health care provide little evidence of any one approach being more effective than another. Trials on the efficacy of occupational health interventions vary in methodological quality, study populations, interventions, reference treatments and outcome measures, leading to a conclusion that no single approach can be favoured over another yet. Some evidence from randomised trials, however, has shown that specific interventions for specific conditions,^12^^–^15 or for selected high-risk groups,^1^ ^2^ may be effective in the occupational health setting. Some of the randomised controlled trials evaluating the effectiveness of specific treatments in the occupational healthcare setting have also included an economic evaluation.^15^^–^17 However, none of the previous studies focusing on high-risk subgroups has carried out such an evaluation. This seems to be the first study to assess cost effectiveness for a specific intervention programme for employees at high risk of sickness absence. However, it is yet to be assessed whether the savings due to the reduction of sickness absence and health resource use in the high risk group exceed the investment in the whole screening process. The cost for the screening for the original 3115 subjects invited was about €46,000.

**Main messages**

- An occupational health intervention, which included an invitation to occupational health service (OHS) for a consultation and, if appropriate, a referral to specialist treatment, was cost saving and more effective in reducing sickness absence than usual occupational health care among employees at high risk of sickness absence.
- The cost effectiveness was not sensitive to any reasonable changes in the values of cost variables.

**Policy implication**

Health check-ups by OHS nurses while targeted for all employees may not be an optimal use of OHS resources. The results of the present study give support for interventions that are targeted to selected employees at a high risk of sickness absence and work disability.

### Meaning of the study

The intervention arm of this study applied a pragmatic approach: the employees’ own occupational nurses and physicians carried out the occupational health intervention. The key difference between the intervention and usual care treatment arms was the invitation to OHS for an additional consultation, and subsequent disease-specific interventions when applicable. With the health survey and subsequent invitation to OHS we succeeded in capturing many workers with underlying health problems that had not been properly attended to. Of the subjects who visited OHS, more than half had not received treatment for the respective health problem at OHS before.^3^

The direct costs related to occupational health care (table 1) did not differ between the groups during the intervention period, which was an unexpected result. We can suggest some possible explanations. First, the OHS personnel had access to a pre-classified and valid biopsychosocial profile while taking care of the subjects in the intervention group. This diminished the need to spend time taking the history, and subsequently the OHS personnel could focus on the overall treatment plan for the underlying health issues. Second, the care path through OHS consultation may have functioned more effectively. 45% of the employees who attended the consultation were referred to a further consultation by a specialist, or to specific interventions. Despite so many referrals to specialist care, the intervention was eventually cost-saving in comparison with usual care. It appears that the subjects in the usual care group used healthcare resources in public or private health care even more. Third, perhaps the intervention group subjects were intervened earlier than the subjects in the usual care group.

The usual care in occupational health in this specific company included, besides medical treatment, regular health check-up visits with OHS nurses, targeted for all employees. However the intervention was superior in controlling sickness absence and reduced healthcare resource use. This indicates that health check-ups by OHS nurses, while targeted at all employees, may not represent an optimal use of scarce OHS resources. Instead, our results support the application of interventions that are targeted at selected employees who are at high risk of sickness absence and work disability.

Our economic evaluation, alongside a pragmatic randomised controlled trial, showed that the invitation of individuals at high risk for sickness absence to a consultation at occupational health care reduces both sickness absence days and healthcare costs while providing the same health outcomes as usual care.

Our results may have implications on OHS policies. Perhaps there should be a shift in the OHS resource use from non-targeted health check-ups towards identifying and intervening the individuals at a high risk of sickness absence and work disability. Future studies in different settings and professional groups are called for.
